# Materials and Structures Inspired by Human Heel Pads for Advanced Biomechanical Function

**DOI:** 10.3390/biomimetics10050267

**Published:** 2025-04-27

**Authors:** Zhiqiang Zhuang, Congtian Gu, Shunlin Li, Hu Shen, Ning Liu, Ziwei Li, Dakai Wang, Cong Wang, Linpeng Liu, Kaixian Ba, Bin Yu, Guoliang Ma

**Affiliations:** 1 State Key Laboratory of Crane Technology, Yanshan University, Qinhuangdao 066104, China; zhuangzq20@mails.jlu.edu.cn (Z.Z.); cg555@sussex.ac.uk (C.G.); shunlin@stumail.ysu.edu.cn (S.L.); shenhu@stumail.ysu.edu.cn (H.S.); liuning23@stumail.ysu.edu.cn (N.L.); ziwei.li@ysu.edu.cn (Z.L.); bkx@ysu.edu.cn (K.B.); yb@ysu.edu.cn (B.Y.); 2 Institute of Mechanical Engineering, Shandong University of Technology, Zibo 266590, China; 3 Key Laboratory of Bionic Engineering, Ministry of Education, Jilin University, Changchun 130022, China; dkwang@jlu.edu.cn; 4 State Key Laboratory of Precision Manufacturing for Extreme Service Performance, College of Mechanical and Electrical Engineering, Central South University, Changsha 410083, China; wangcong@csu.edu.cn (C.W.); linpengliu@csu.edu.cn (L.L.)

**Keywords:** heel pad, structure, materials, biomechanical function

## Abstract

The heel pad, located under the calcaneus of the human foot, is a hidden treasure that has been subjected to harsh mechanical conditions such as impact, vibration, and cyclic loading. This has resulted in a unique compartment structure and material composition, endowed with advanced biomechanical functions including cushioning, vibration reduction, fatigue resistance, and touchdown stability, making it an ideal natural bionic prototype in the field of bionic materials. It has been shown that the highly specialized structure and material composition of the heel pad endows it with biomechanical properties such as hyperelasticity, viscoelasticity, and mechanical anisotropy. These complex biomechanical properties underpin its advanced functions. Although it is known that these properties interact with each other, the detailed influence mechanism remains unclear, which restricts its application as a bionic prototype in the field of bionic materials. Therefore, this study provides a comprehensive review of the structure, materials, biomechanical properties, and functions of the heel pad. It focuses on elucidating the relationships between the structure, materials, biomechanical properties, and functions of heel pads and proposes insights for the study of bionic materials using the heel pad as a bionic prototype. Finally, a research idea to analyze the advanced mechanical properties of heel pads by integrating sophisticated technologies is proposed, aiming to provide directions for further in-depth research on heel pads and inspiration for the innovative design of advanced bionic materials.

## 1. Introduction

The human foot possesses a highly intricate anatomical structure and fulfills essential biomechanical functions [1]. During standing and locomotion, it endures substantial loads and impact forces, which are effectively dissipated through the integrated action of skeletal elements, articulations, musculature, and soft tissues. This coordinated mechanism not only facilitates postural balance and stability but also ensures the efficiency and fluidity of gait [2,3]. The foot’s roles in weight-bearing, dynamic support, and adaptability are critically dependent on the structural and functional integrity of its soft tissue components [4].

The heel pad, one of the most important soft tissues of the foot, is a highly specialized biological tissue in terms of structure and material composition, located beneath the calcaneus [5,6,7]. It is the first tissue to contact the ground during walking and is subjected to intense mechanical forces. During vigorous exercise, the impact force on the heel can reach 2–3 times the body weight [8]. Despite the human body taking tens of thousands of steps per day, the heel pad maintains its structural integrity and stable mechanical properties [9]. These extreme mechanical conditions have resulted in the heel pad evolving a highly specialized compartmental structure and material composition. Its biomechanical properties include flexibility, damping, rigidity, non-homogeneity, and mechanical anisotropy. These properties enable the heel pad to perform critical biomechanical functions such as cushioning [10], vibration damping [11], anti-fatigue [12], and providing ground stability. As a result, the heel pad is a multifunctional biological tissue with advanced mechanical properties, making it an ideal prototype for bionic functional materials.

However, the interaction mechanisms between the compartmental structure, material composition, biomechanical properties, and biomechanical functions remain unclear. This lack of understanding limits the application of the heel pad as a bionic prototype in the development of advanced bionic materials. Analyzing the mechanisms underlying these interactions could provide critical insights, promoting the use of the heel pad as a model for creating bionic materials with superior mechanical properties.

**Figure 1:**
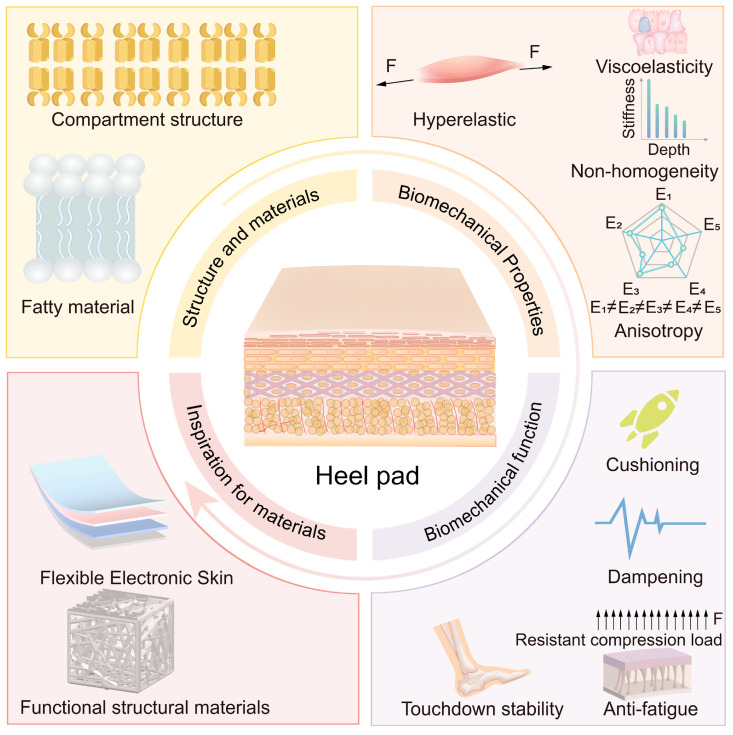
The overall research line of this study. The research line includes four aspects: compartment structure and material properties, biomechanical properties, biomechanical function, and inspiration for developing advanced materials.

Therefore, this study investigates the compartmental structure and material composition of the heel pad, analyzes their impact on biomechanical properties, and elucidates how these properties determine its functional performance. Furthermore, the heel pad is explored as a bionic prototype to inspire the design of advanced biomimetic materials. The overall research framework is presented in Figure 1. By clarifying the structure–property–function relationship, this study aims to inform future heel pad research and contribute to the development of innovative bionic materials.

## 2. Structure and Materials of Heel Pad

### Structure of the Heel Pad

**Figure 2:**
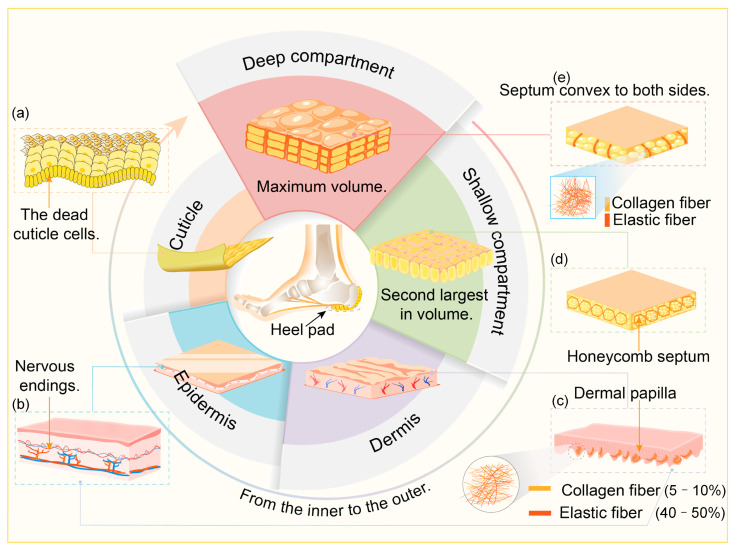
Structure of the layers of the heel pad. (**a**) Cuticle. (**b**) Epidermis. (**c**) Dermis. (**d**) Shallow compartments. (**e**) Deep compartments.

The heel pad, located beneath the calcaneus, has an overall thickness of approximately 14–19 mm and consists of five layers from the outermost to the innermost layer, the cuticle, epidermis, dermis, shallow compartment, and deep compartment [5,13,14], as illustrated in Figure 2. Among these, the deep compartment is the thickest (about 12–18 mm), accounting for the largest volume [15], and serves as the primary functional layer responsible for the heel pad’s biomechanical properties. The shallow compartment, approximately 2–3 mm thick [6], lies above the deep compartment, while the cuticle, epidermis, and dermis are relatively thin.

The cuticle is located in the superficial layer of the heel pad, which is the external-most layer of the skin of the foot [16]. The cuticle of the plantar skin is thicker than that of other body parts [17], as shown in schematic Figure 2a. It consists of dead keratinocytes and has the primary function of protecting the heel skin from external irritation and water evaporation. Due to bearing the weight of the body and often with ground friction, the heel pad of the cuticle layer is relatively thickened, and the cuticle layer can reduce pressure on the foot by increasing the friction between the heel pad and the ground, which has a certain anti-slip function [18].

The epidermis is located under the cuticle, and this layer is firmly attached to the surface of the dermal papillae, as shown in schematic Figure 2 [16]. The epidermis maintains the skin’s water balance by regulating skin moisture through the stratum corneum’s keratinous scale structure and the lipid layer between keratinocytes. The epidermis of the heel pad contains a large number of sensory nerve endings, enabling the skin to perceive and transmit sensory information such as touch, temperature, and pain [19], as shown in Figure 2b. At the same time, this layer has the effect of maintaining body temperature by regulating the blood flow of the skin and the secretion of sweat glands, which help to dissipate excess heat.

**Figure 3:**
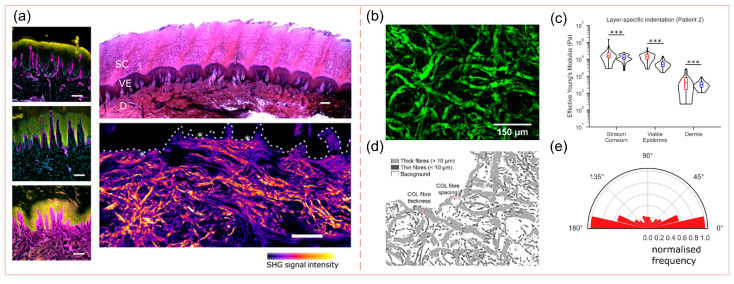
Microstructural characterization of the various layers of tissue in the heel pad. Reproduced with permission from Ref. [20], Copyright 2019, Science Advances, The American Association for the Advancement of Science. (**a**) Cross-structure of the epidermis and dermis. (**b**) Collagen fiber image in dermal papillae. (**c**) Young’s modulus of the epidermis and dermis. (**d**) SHG segmentation image showing thick (grey) and thin (dark grey) collagen fibers. (**e**) The collagen fibers in the dermal papillae are oriented so that 90% of the collagen fibers are at 180° to the horizontal. *** *p* < 0.001, two-sided Student’s *t* test.

The dermis, situated beneath the epidermis, contains dense papillary structures rich in fine collagen and elastic fibers. These fibers interweave randomly to form fiber bundles, with collagen accounting for 5–10% and elastic fibers for 40–50% [6,20], and the elastic modulus of the epidermis is slightly higher compared to the dermis, which is 0.12MPa, as shown in Figure 2c. There is a large cross-structure at the junction of the epidermis and the dermis [21], as shown in Figure 3a, and the modulus of the epidermis is slightly higher compared to the dermis, as shown in Figure 3c. Compared with non-plantar skin, collagen fibers in the dermis of the heel pad are arranged in coarser bundles, as shown in Figure 3b,d, and the percentage of parallel bundles of coarser fibers is higher [21], as shown in Figure 3e, which could improve the resistance of plantar skin to shear and tearing, thus protecting plantar skin from large deformation injuries, such as pressure ulcers [21].

The shallow compartment layer is located in the middle of the dermis and the deep compartment, which contains equal amounts of collagen fibers and elastic fibers [6,15]. The collagen fibers and elastic fibers are interwoven into septa, which are interwoven into a honeycomb structure, and the interior of the honeycomb compartments is wrapped with fine fatty globules [15,22], as shown in Figure 2d; the fatty globules are enriched with lipid droplets, whose main component is triglycerides. The ink-printing test studies showed that the honeycomb compartments were independent of each other and were not connected to each other [20]. Tested by the ultrasound system, the modulus of elasticity of the shallow compartments is 450 kPa, which is ten times that of the deep compartments, and its main function is to prevent excessive deformation of the heel pad [14].

The deep compartment layer is located in the innermost layer of the heel pad, which is rich in collagen fibers and elastic fibers, with 50–60% elastic fibers [20], as shown in Figure 2e. The fibrous septa of this layer have a highly specialized structure; in the sagittal plane, the fibrous septa are interwoven into U-shaped or comma-shaped compartments, with the open ends facing the calcaneus [7,15,23], and in the coronal plane, the fibrous septa are curved laterally [15]. Nuclear magnetic imaging showed that the fibrous septa on the coronal plane were convex to both sides in a crescent-shaped structure, and the septa were convex outward to each side under compressive loading [24]. The deep compartments are similarly independent of each other and contain even smaller compartments, which are wrapped with fat mass inside. Ultrasound test have shown that the elastic modulus of the deep compartment is 46 kPa [14], which provides the heel pad with sufficient elasticity, viscosity, and compression resistance, and this layer determines the biomechanical functions of the heel pad such as cushioning, vibration damping, and mechanical anisotropy.

In summary, this paper shows that the current structural characterization of heel pads is limited to a two-dimensional plane. Therefore, in future characterization studies of the heel pad compartment, its compartment structure can be scanned by advanced imaging techniques, such as Micro-CT and Micro-MR, to establish a three-dimensional model of its compartment structure, which can provide a theoretical model for the bionic design of advanced materials.

The heel pad also contains nerves that are separate from the surrounding tissue as well as a blood supply system. There are two main branches of nerves in the heel pad, which penetrate the entire heel pad, and free nerve endings and pacinian corpuscles are located inside the heel pad, where sensory nerves on both the inner and outer sides enter the forefoot fat pad [15,19].

In the adipose tissue of the heel pad, the lipid droplets in the adipocytes make up 95% of the entire cell volume and are mainly composed of triglycerides, which are a low-viscosity liquid [25]. Compared to fatty tissue in other parts of the body, the heel pad contains 19–25% more unsaturated fatty acids, with an unsaturated-to-saturated ratio of 4.4 [24]. The high ratio of unsaturated to saturated fatty acids means that heel pad is less viscous; this is often simplified to a non-sticky liquid [26,27]. This structure and material composition can provide an important reference value for bionic functional materials. However, there is still a lack of micro-characterization of the structure of the septum, such as what is the distribution pattern of collagen fibers and elastic fibers within the ratio, what is the content of collagen fibers and elastic fibers, and what is the content of fat in the heel pads, which are still not clear. These problems might be solved by reverse engineering modeling of the heel pad.

## 3. Biomechanical Properties of Heel Pad

### 3.1. Nonlinear Stiffness (Hyperelasticity)

**Figure 4:**
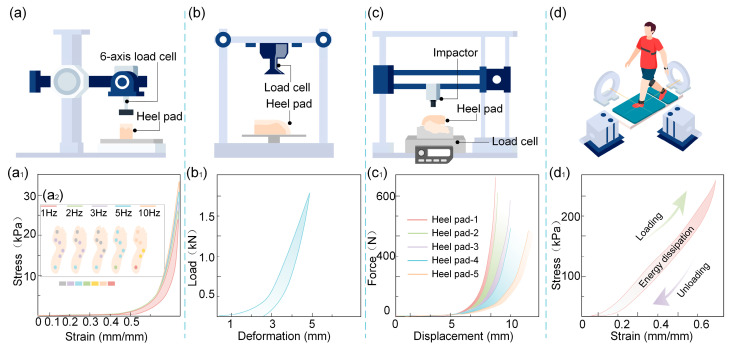
Test methods for studying the stiffness of the heel pad. (**a**) Test setup for compression of in the vitro heel pad without lateral limit. (**a1**) Stress–strain curves of the in the vitro heel pad. (**a2**) Effect of test frequency on the stiffness of the heel pad. (**b**) Test setup for in vitro test study. (**b1**) Stress–strain curves of the heel pad in in vitro test. (**c**) Impact test platform for in the vitro heel pad with calcaneus. (**c1**) Load–displacement curve for the heel pad in the state of impact. (**d**) In vivo test of the heel pad. (**d1**) Load–displacement curve of the heel pad in vivo test.

The nonlinear stiffness of the heel pad refers to the nonlinearity of its load–displacement curve under compressive loading [28,29]. The research methods mainly include in vitro tests with the heel pad [30], in vitro tests without the heel pad [31], and in vivo tests [32], as shown in Figure 4. The in vitro test is mainly performed using a universal testing machine or impact testing machine [4], as shown in Figure 4a–c. The heel pad under compressive loading, both with and without the calcaneus, is mechanically characterized by nonlinearity of stiffness, as shown in Figure 4(a1–c1). In these different tests, Figure 4b,c show heel pads with the calcaneus, and Figure 4a shows heel pad samples without the calcaneus. For the in the vitro heel pad, at a compression load of twice the body weight, the compression is 4–7 mm and the load–displacement curve is nonlinear; as the compression load increases, the slope of the load–displacement curve increases, i.e., the stiffness of the heel pad increases [30]. It was shown that this nonlinear mechanical characteristic plays an important function in the touchdown stability of the heel pad [4]. With the increase in the test frequency of the compression heel pad, its stiffness gradually increases. Based on this, Ker investigated the effect of intermittent cyclic loading on the mechanical properties of the heel pad using an in vitro test, and the test results showed that the shorter the interval between adjacent cyclic loads, the higher the stiffness of the heel pad. In addition, the stiffness of the heel pad is also affected by the test frequency. The higher the test frequency, the higher the stiffness of the heel pad [29], as shown in Figure 4(a2).

In vivo testing of the heel pad is primarily conducted using a force plate and a biplane X-ray imaging system [32], as shown in Figure 4d. During human walking, the heel pad continues to exhibit nonlinear mechanical behavior, as illustrated in Figure 4(d1). The stiffness of the heel pad in the in vivo state is affected by the impact velocity and the test frequency; the faster the impact velocity and the higher the test frequency, the higher the stiffness [33,34]. In addition, heel pad stiffness is also affected by age, as studies have shown that the stiffness of the heel pads of the elderly is higher than that of the heel pads of young people [35,36]. The increased stiffness of the heel pad in older adults may result from age-related degeneration, including collagen loss, reduced elastic fibers, and decreased elasticity of the fibrous septa, which often become thickened and rigid. The heel pad lesions can also lead to septal thickening and hardening, further exacerbating its nonlinear stiffness [28,37,38]. In addition, the volume fraction of fibers in the heel pad as well as the orientation also have an important effect on the nonlinear stiffness of the heel pad [13].

Another manifestation of nonlinear stiffness is nonlinear modulus [39]. Currently, more research reports focus on characterizing the relationship between stress and strain in the heel pad, and the modulus of the heel pad tends to increase with increasing compressive load [40]. In this study, it was found that the modulus of the heel pad differed greatly under different test conditions. For example, the ultrasound-measured modulus of the heel pad at 50% deformation was 600 kPa [41], while the modulus at 50% deformation was 2 MPa when the heel pad was compressed at 1 mm/s [38]. Miller-Young cut the heel pad into cylinders with a diameter of 8 mm for unconfined compression, and the modulus of the heel pad at 50% deformation at a compression speed of 175 mm/s was only 18.4 kPa [31]. It can be visualized through Figure 4(a1,d1) that at the same strain, the stress in the heel pad in vivo is higher than in vitro. This study suggests that the reason for the significant difference in the results above may be due to the integrity of the tissue of the in vitro heel pad, which is prone to destruction of the compartment structure after being cut, and the compression process is prone to fat spillage; thus, the modulus of the heel pad in vitro is lower during compression.

The relationship between the stress and strain of the heel pad is nonlinear and does not satisfy the theory of linear elastic deformation. Therefore, the heel pad, as a hyperelastic material, can be modeled using an energy density function—typically dependent on strain and incorporating linear, nonlinear, and dissipative terms—to predict its stress–strain behavior under various loading conditions [42,43,44,45,46,47].

To describe the relationship between stress and strain in the heel pad, the following hyperelastic mathematical models are commonly used in the literature, with specific parameters shown in Table 1: Ogden, Mooney-Rivlin, Generalized Rivlin, and polynomials.

The compartment structure and material composition of the heel pad determine its nonlinear stiffness [48]. Since the heel pad compartments are independent of each other, the fat in liquid state constitutes a separate hydrostatic unit within the compartments [20]. Under pressure, the fat expands around the periphery, which leads to the stretching of the septum, and consequently the fibers within the septum are also stretched. The reticular fibrous septum consists of collagen fibers and elastic fibers, which have a spiral or wavy structure [49]. In the early stage of compression, the collagen fibers and elastic fibers within the septum are in a relaxed wave-like structure, and the deformation of the septum requires a smaller load, so the heel pad can undergo a larger deformation under a smaller compression load, and the stiffness is smaller at this time. As the compression load increases, the collagen fibers in the septum are gradually straightened, and the stiffness of the heel pad continues to increase [35]. As the compressive load continues to increase, the collagen fibers become progressively straightened and further stretched. During this phase, even minor deformations of the heel pad require substantial compressive loads, indicating a marked increase in heel pad stiffness in the later stages of compression. The fibrous septum consists of collagen fibers and elastic fibers. Elastic fibers are flexible and have low stiffness, while collagen fibers have high stiffness, and most of the load on the heel pad is carried by the collagen fibers. The main role of the elastic fibers is that when the compression load disappears, it pulls the straightened collagen fibers back to their initial state [27].

### 3.2. Viscoelasticity

**Figure 5:**
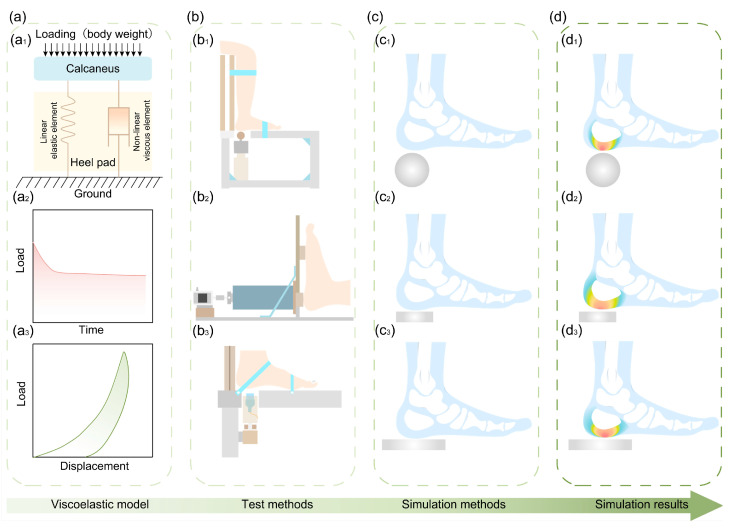
Test method and finite element (FE) simulation model for viscoelastic mathematical modeling of heel pad. (**a**) Viscoelastic model and mechanical curves of heel pad. (**a1**) Viscoelastic model. (**a2**) Load relaxation curve. (**a3**) Hysteresis curve. (**b**) In vivo viscoelasticity test method. (**b1**) Spherical head indentation test. (**b2**) Disk indentation test. (**b3**) Ultrasonic probe indentation test. (**c**) Simulation validation of heel pad viscoelasticity. (**c1**) Ball indentation test simulation. (**c2**) Disc indentation test simulation. (**c3**) Platform indentation test simulation. (**d**) Finite element simulation results of heel pad. (**d1**) Scheme of ball indentation test simulation result. (**d2**) Scheme of disc indentation test simulation result. (**d3**) Scheme of platform indentation test simulation result.

Viscoelasticity means that a material has both viscous and elastic properties. In viscoelastic materials, there is a delay effect between the rate of change of strain with time and stress [47,50,51,52]. A mathematical model of viscoelasticity is shown in Figure 5(a1). Viscoelastic materials tend to possess the following three mechanical characteristics: creep, stress relaxation, and hysteresis [53,54,55,56]. Similar to other biological soft tissues, the heel pad is not simply an elastic material but a material that is both viscous and elastic, with stress relaxation, creep, and hysteresis. Stress relaxation refers to the phenomenon of gradual reduction in stress within a material under constant strain conditions, and similar to creep, stress relaxation is a time-dependent change in the material [57], as shown in Figure 5(a2). Relevant studies have shown that an initial load of 15 N on the heel pad was reduced by 40% after 300 s while keeping the displacement constant [29], and the material composition and structure might be the reason for the rapid stress relaxation that occurred in the heel pad, where the low viscosity of the lipids in the heel pad allowed for faster dispersion and release of the stresses, which led to the rapid stress relaxation [31].

At present, most studies are devoted to characterizing the viscous characteristics of plantar soft tissues and establishing mathematical models that can accurately respond to the viscoelasticity of the heel pad [58,59,60], with the method shown in Figure 5. Firstly, a suitable viscoelastic model for the heel pad is analyzed and selected. The corresponding mathematical formulation is shown in Figure 5(a1). Next, viscoelastic testing of the heel pad is conducted. In vivo indentation tests mainly involve devices such as a ball head, ultrasonic probe, and disc, as illustrated in Figure 5(b1–b3). The viscoelastic mathematical model is then used to fit the test data in order to obtain the model parameters. Based on the chosen testing method, a corresponding finite element model is established, as shown in Figure 5(c1–c3). Finally, simulation is performed for model verification, as illustrated in Figure 5(d1–d3).

**Table 1:** Hyperelastic model and parameters of heel pad.

Hyperelasticity Model	Parameters	
Ogden [47,61]	$\mu \left(MPa\right)=0.036\;\alpha =4.5$	$\mu \left(MPa\right)=0.016\;\alpha =6.82$
Mooney-Rivlin [45]	$C_{10}\left(MPa\right)=-0.000871$ $C_{01}\left(MPa\right)=0.00405$	$C_{10}\left(MPa\right)=0.000449$ $C_{01}\left(MPa\right)=0.00429$
Generalized Rivlin [45]	$C_{10}\left(MPa\right)=0.00395$ $C_{20}\left(MPa\right)=0.000703$ $C_{30}\left(MPa\right)=0.00134$	$C_{10}\left(MPa\right)=0.00572$ $C_{20}\left(MPa\right)=0$ $C_{30}\left(MPa\right)=0.00240$
Polynomial [41]	$C_{10}\left(MPa\right)=0.85550\;C_{20}\left(MPa\right)=-0.584$ $C_{30}\left(MPa\right)=0.3892$ $C_{11}\left(MPa\right)=-0.231$ $C_{02}\left(MPa\right)=0.08484$ $D_{1}\left(MPa\right)=0.00000437$ $D_{2}\left(MPa\right)=0.00000068$	
Hyperelastic constitutive model is defined by the strain energy function [62]	Fat: $K_{v}\left(MPa\right)=2.31\times 10^{-1}$ $r=2.74\times 10^{1}$ $C_{1}\left(MPa\right)=2.91\times 10^{-3}\;\alpha_{1}=5.35\times 10^{1}$ Collagen fiber: $K_{v}\left(MPa\right)=2.02\times 10^{-2}$ $C_{1}\left(MPa\right)=4.63\times 10^{-3}$ $C_{4}\left(MPa\right)=2.36\times 10^{-1}\;\alpha_{4}=5.48\times 10^{1}$	

Relevant studies have shown that the Maxwell model can better fit the load–relaxation curve generated by the ball indentation heel pad [63], and the specific parameters are shown in Table 2. Based on this Maxwell model, Takuo Negishi again verified the validity of the spherical indentation method for detecting the viscoelasticity of heel pad, and he reproduced the load–relaxation curves of heel pad at different strain rates by employing a fifth-order Maxwell model [64]. In addition, in the indentation test of the heel pad with pistons of different diameters, as shown in Figure 5(b2), the higher energy loss rate is due to the fact that the large-diameter piston compresses the heel pad to respond to their overall mechanical properties, while the small-diameter piston only responds to the localized mechanical properties of the heel pad, and thus the energy loss rate is lower [65]. Considering that the arrangement of collagen fibers and the movement of liquid components inside the heel pad may cause changes in the viscoelastic parameters, by setting the viscous variables related to the arrangement of liquid components and collagen fibers inside the heel pad, A. N. Natali developed a visco-hyperelasticity constitutive model, which is able to explain a variety of mechanical properties of the heel pad, such as geometric nonlinearity, almost incompressibility and time dependence, etc., and the results of stress relaxation tests are in good agreement with the results predicted by this model [52].

Hysteresis is a phenomenon in which the relationship between the strain and stress of a material does not exactly coincide during loading and unloading [68,69], as shown in Figure 5(a3). The strain of a viscoelastic material during unloading is not exactly the same as the strain during previous loading. It has been shown that the heel pad has significant hysteresis under compressive loading [70].

The two load–displacement curves of the heel pad do not coincide during loading and unloading, forming a closed hysteresis loop. Since the area of the hysteresis loop indicates the energy lost by the heel pad during loading and unloading, the energy loss rate of the in vitro heel pad was calculated to be 28.6 ± 6.9% [30].

**Figure 6:**
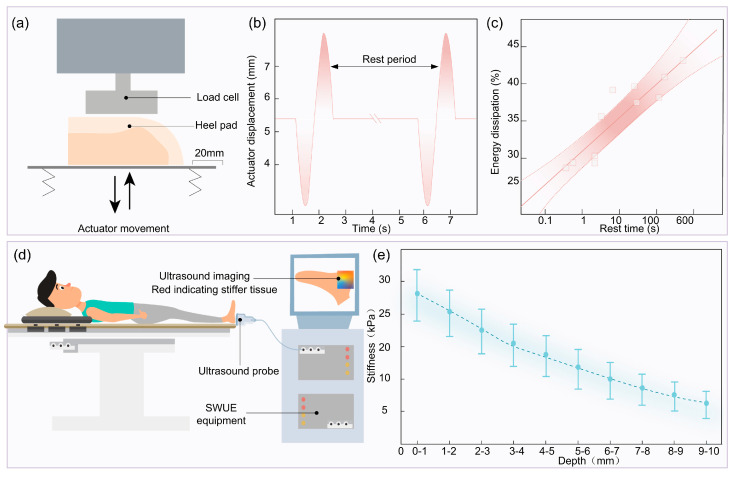
(**a**) Experiments on the effect of time intervals on the hysteresis mechanical properties of heel pads. (**b**) The movement pattern of the heel pad compressed by the actuator. (**c**) Effect of interval time on the rate of energy loss of the heel pad. (**d**) Test the stiffness of each layer of the heel pad using SWUE. (**e**) Trend of heel pad stiffness with increasing depth from the epidermis to the internal compartments.

However, the frequency has less effect on the hysteresis of the heel pad. On this basis, Aerts studied the effects of different cyclic compression loads and different time intervals on the energy loss of the heel pad by applying loads to the in vitro heel pad at certain time intervals. As shown in Figure 6a,b, the experimental results showed that the energy loss rate of the first compression of the heel pad was 17% higher compared with the energy loss rate after the nth cycle of compression. Further, Robert Ker investigated the effect of compression time interval on the hysteresis characteristics of the heel pad. The energy loss rate of the heel pad was 33% when the interval time was 1s, the energy loss rate of the heel pad increased by 3.7% for every 10 times increase of the interval time, and the energy loss rate and the logarithm of the interval time had a linear relationship, as shown in Figure 6c.

The method of numerically integrating the area below the stress–strain curve during loading and unloading of the heel pad, calculating the energy input density and energy return density, and then fitting the energy density–strain data to the energy function of a nonlinear viscoelastic model allows for an accurate description of the hysteresis properties of the heel pad, and, at the same time, it allows for the quantification of the elastic and viscous parameters that characterize the hysteresis properties of the heel pad [43]. Based on this, C.G. Fontanella considered the nonlinear, almost incompressible, and time-varying properties of the heel pad, and developed a visco-hyperelastic constitutive model capable of responding to these properties [71]. This model was validated to reproduce the characteristics of the hysteresis return of the heel pad and accurately reflect the energy loss rate of the heel pad. In addition, the Yeoh model [66], the Ogden model [72], and the first-order Maxwell model were able to accurately respond to the mathematical model of the viscoelasticity of the heel pad; for the hysteresis characteristics of the heel pad, the predictions of these models and the experimental results were in excellent accordance.

Creep is the time-dependent deformation of a material that gradually extends or deforms with time under constant stress [73,74]. Until now, in the research related to the mechanical properties of heel pads, no research report on the creep properties of heel pads has been found. The understanding of viscoelasticity of heel pads can be deepened in future studies by carrying out relevant studies on in vitro heel pads and in vivo heel pads.

There are too many different views on the reasons for the viscoelasticity of biological tissues. Synchrotron X-rays combined with mechanical tests have shown that collagen fibers, the main component of biological soft tissues, are viscoelastic themselves, and that collagen fibers and the surrounding proteoglycan-rich matrix together determine the viscoelasticity of biological tissues [75]. However, more studies have shown that the proteoglycan-rich matrix surrounding collagen fibers is a major contributor to the viscoelasticity of biological soft tissues [76], and researchers such as Fung, Thornton, and Gupta have argued that the different compositions of the tissues and their structural arrangements give rise to different viscoelastic properties, and that the aggregation of collagen fibers controls the creeping behavior of biological soft tissues, whereas collagen fibers slipping through proteoglycan-rich matrices control the relaxation behavior of tissues [77,78]. In summary, the main reasons for the viscoelasticity of heel pads may include the following two aspects: the first is that the liquid components in the heel pad flow in the compartment to generate friction resistance, and the viscoelasticity is caused by friction resistance; the second is that the interaction between the matrix and collagen fibers in the heel pad makes them have viscoelasticity.

### 3.3. Non-Homogeneity

Non-homogeneity of biological tissues refers to the inhomogeneity of tissue structure and properties in living organisms [79,80]. Biological tissues are usually composed of different types of cells, extracellular matrix, and other components, which differ in spatial distribution, organizational structure, and other aspects. At the same time, the non-homogeneity of biological tissues is also reflected in the functional differences of the tissues, and different tissues have unique characteristics in morphology, structure, and function, such as the contraction ability of muscle tissues, the conduction ability of nerve tissues, and the supportive function of skeletal tissues [81].

Shear wave ultrasound elastography (SWUE) allows for noninvasive and quantitative assessment of soft tissue elasticity, and since the region of interest can be located at different depths, SWUE allows for individual assessment of the stiffness of different layers of the heel pad [82,83]. An ultrasonic test study showed that the stiffness cloud color of the shallow compartments was significantly darker than that of the deep compartments, as shown in Figure 6d. From the outer epidermis to the inner deep compartments, the stiffness of the heel pad decreases with increasing depth, as shown in Figure 6e, i.e., the stiffness of the epidermis of the heel pad is greater than that of the shallow compartments, and the stiffness of the shallow compartments is greater than that of the deep compartments [84]. This indicates that the heel pad has different stiffnesses in different regions, possessing non-homogenous properties.

In the heel pad, the size of the fibrous compartments, the thickness of the septum, and the amount of fatty tissue were different at different locations, and these factors made the mechanical properties of the heel pad non-homogeneous and mechanically anisotropic [23]. In addition, the collagen fibers in the heel pad are directional [85,86], which causes the mechanical response along the direction of the fibers to be significantly different from that perpendicular to the fibers, which may be another reason for the non-homogeneity of the heel pad.

### 3.4. Mechanical Anisotropy

Mechanical anisotropy refers to the variation of material properties in different directions, a characteristic common in most biological tissues [87,88]. Directional differences in physical, chemical, or mechanical properties exist at both the micro and macro levels. Microscopically, cellular morphology and arrangement may vary with direction [89,90], while macroscopically, tissue organization reflects anisotropic behavior. For example, the bone trabeculae of skeletal tissues shows orientation-dependent differences in density and structure, leading to varying strength and stiffness [84], and the fiber alignment in muscle tissue influences its mechanical response and contraction direction [91,92].

**Figure 7:**
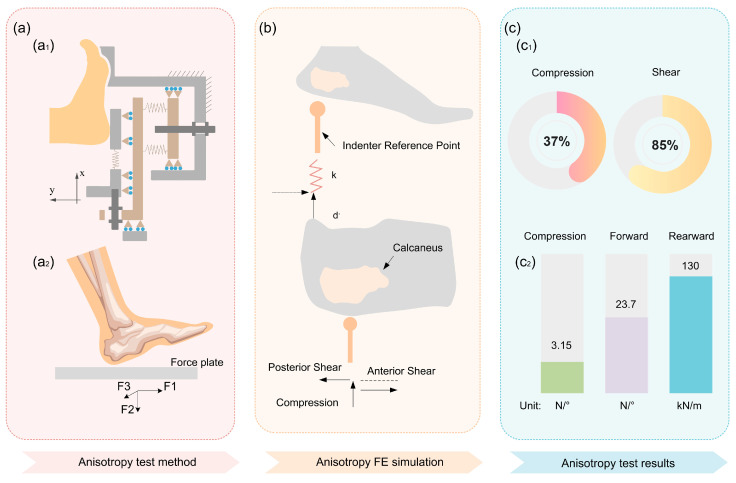
Summary of the current status of research on the mechanical anisotropy of the heel pad. (**a**) Anisotropic test setup. (**a1**) Self-constructed test setup. (**a2**) Force plate. (**b**) FE simulation of the mechanical anisotropy of the heel pad. (**c**) Results of the mechanical anisotropy test of the heel pad. (**c1**) Energy dissipation of the heel pad in different directions. (**c2**) Stiffness of the heel pad in different directions.

Similar to most biological tissues, the heel pad also has mechanical anisotropy. The main test devices for testing the mechanical anisotropy of the heel pad is self-built test equipment [93], as shown in Figure 7(a1); force plate-infrared light cameras [94], as shown in Figure 7(a2); and robotic arms [42]. The mechanical characteristics of the in vitro heel pad in different directions have been tested using a six-degree-of-freedom robotic arm and verified using FE simulation in the way shown in Figure 7b. The test and simulation results show the three load–displacement curves of the heel pad measured in the three directions of vertical compression, anterior, posterior, and shear. The test and simulation results show that the three load–displacement curves of the heel pad measured in the three directions of vertical compression, anterior-posterior shear, and left-right lateral shear are significantly nonlinear, and the three curves do not overlap with each other, which satisfies the characteristics of mechanical anisotropy.

The mechanical anisotropy of biological tissues is often related to the structure. For example, the arrangement and distribution of collagen fibers in muscle tissue is the main reason for its mechanical anisotropy [95]. In the longitudinal tensile direction, muscles usually have higher tension and tensile strength and are able to produce larger pulling force, while in the transverse shear direction, the mechanical properties of muscles are relatively weak. Similarly, the mechanical anisotropy of heel pads may be determined by the structure of their compartments, and the shape and structure of the compartments and the distribution of the septum may be the main reasons for the differences in the mechanical properties of the heel pad in different directions.

Specifically, the hyperelasticity, viscoelasticity, and mechanical anisotropy of the heel fat pad show a complex coupling relationship under different loading conditions. Hyperelasticity mainly reflects the nonlinear response of the tissue under large deformation conditions, viscoelasticity embodies its response to time-dependent loads (e.g., sustained compression or cyclic impact), and mechanical anisotropy originates from the differences in fiber orientation within the tissue, resulting in differences in mechanical properties in different directions. Under fast impact loading, hyperelasticity dominates the overall response, anisotropy determines the local deformation path, and viscoelasticity plays a relatively small role. Under sustained or low-frequency loading, viscoelastic deformation gradually increases, fluid migration and tissue creep are obvious in vivo, and the coupling of hyperelasticity and anisotropy affects the energy dissipation and stress distribution path. In addition, the anisotropic structure modulates the local stiffness distribution of the tissue, thus affecting the deformation and viscoelastic energy dissipation process in the hyperelastic region at the microscopic level.

## 4. Biomechanical Function of Heel Pad

### 4.1. Cushioning

**Figure 8:**
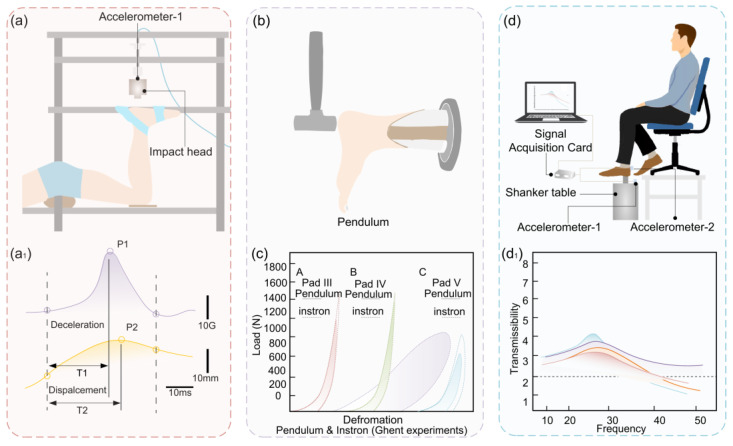
Cushioning and vibration damping tests of the heel pad. (**a**) Falling hammer cushioning test of the heel pad. (**a1**) Acceleration of a falling hammer on the heel pad. (**b**) Pendulum cushioning test of the heel pad. (**c**) Comparison of the energy loss rate of the heel pad under different test conditions. (**d**) Vibration damping test of the heel pad. (**d1**) Transmissibility–frequency curves of the heel pad.

During walking or running, the heel pad is always subjected to different degrees of impact, and large impact loads often cause serious injuries to the human body. In order to protect the human body, the heel pad exhibits good cushioning performance [70,96]. In the body impact test, as shown in Figure 8a, the maximum deformation of the heel pad is 11.3 mm and the maximum peak acceleration is 11.6 G when the heel pad is impacted at 0.93 m/s, and the peak acceleration of the sole is 13.8 G at the same speed, and the heel pad has good cushioning performance compared with that of the sports shoes [97], and the result of the test is shown in Figure 8(a1).

When subjected to compressive loads, the hyperelastic heel pad undergoes large deformations, the maximum deformation of which can exceed 60% of the total thickness of the heel pad, with excellent flexibility [64]. When touching the ground, its flexibility allows the foot to decelerate over a certain distance, which prolongs the acceleration time and reduces the peak acceleration of the foot, thus reducing the peak impact force. The heel pad protects the foot and limb from impact injuries by reducing the peak impact force to a range that the foot’s skeletal and muscular systems can withstand. At the same time, the compartment in the heel pad spreads the impact load over the entire plantar surface, reducing contact stresses by increasing the force area [6].

The cushioning performance of the heel pad is influenced by various factors, with the compartment structure being a key determinant. Compared with thin heel pads, thicker heel pads can extend the distance of heel deceleration and have good cushioning performance [6]. In addition, the stability of the fixation of the upper and lower ends of the septum affects the heel pad deformation capacity. If the joint between the compartment and the surface of the calcaneus is broken, the entire heel pad is more likely to bulge out to the periphery, which results in a thinner heel pad at the mid-position and thus worse cushioning performance. In addition, age is also an important factor affecting the cushioning performance of heel pads [97]. In the heel pad of the elderly, the aging of the compartment structure has resulted in the loss of collagen in the septum, the reduction of elastic fibers and water content, the thickening of the septum, and the occurrence of local ruptures of its internal fibrous tissues, which has led to the hardening of the heel pad, the deterioration of flexibility, and consequently to the deterioration of its cushioning performance [36]. In addition, lesions are also a major factor affecting the cushioning performance of the heel pad [37,98]. Due to the lesion of the heel pad, the internal fiber septum becomes thicker and hardes [99,100,101], and the hardened heel pad becomes less flexible and loses elasticity, which leads to the deterioration of its cushioning performance. In summary, the elastic fiber compartments and their wrapped viscoelastic fat in the heel pad provide flexibility, and the heel pad undergoes large deformation after an impact load, absorbing a large amount of impact energy and thus providing excellent cushioning performance. In addition, the higher number of compartments and the lower fat content may cause the heel pad to be too hard and poorly flexible. Therefore, the number of compartments and the fat content in the heel pad may be in a specific range, and the coupling of the two results in excellent energy absorption.

### 4.2. Vibration Damping

Vibration is prevalent in daily life and work, with the human body frequently exposed to ground vibrations during walking, running, and jumping. Harmful vibrations can pose significant risks to human health and safety [102]. In order to reduce the vibration damage to the human body, the organizational structure and material properties of the heel pad play a good role in absorbing, attenuating, or transferring vibration.

Vibration damping performance is related to the energy dissipation during the deformation of the heel pad, and due to viscoelasticity, the energy dissipated by the in vitro heel pad under compressive loading is about 30% [30]. In the in vivo drop hammer test, the heel pad showed excellent flexibility, and its energy absorption rate was as high as 75–80% [36].

The energy loss rates of the heel pad under different test conditions are shown in Table 3, which shows that there is a large difference in the energy dissipation rates of the heel pad in in vivo and vitro tests. It is believed that the reason for this difference is related to the test conditions. In the body drop hammer test, the impact energy can be absorbed by several tissues such as the heel pad, the cartilage of the ankle and talar joints, and the soft tissues around the knee joint [35]. The energy loss rate measured in the in vitro test only reflects the performance of the heel pad itself. The in vitro test with calf and heel pad verified the above speculation that the soft tissues inside the calf have a role in energy loss, as shown in Figure 8b, and the presence of the calf is the reason for the high energy loss rate of the in vivo heel pad, and the results are shown in Figure 8c [103]. In addition, relevant studies have also tested the attenuation effect of the foot on vibration waves using shakers and accelerometers, as shown in Figure 8d, and the results show that the resonance frequency of the heel pad is in the range of 20–35 Hz [104], which dissipates more applied vibration energy than the talocrural joints, and it has a good vibration damping effect, and the results are shown in Figure 8(d1).

The structure of the heel pad and its material properties provide excellent vibration damping properties. In the heel pad, the reticular fiber septum is interwoven into individual compartments, and the liquid fat is filled in each compartment chamber, thus forming a large number of hydrostatic units. The hydrostatic unit provides excellent energy absorption and vibration damping, which contributes to the heel pad’s effective vibration-damping performance. Due to the viscoelasticity of the heel pad, the energy absorption of the heel pad could dissipate in the form of viscoelastic creep, heat, etc., thus weakening the vibration. Additionally, the fiber septum and fatty unit that make up the heel pad act as two coupled elements with vibration damping and energy storage functions. These elements are cross-coupled, allowing them to work together effectively to dampen vibrations during human movement [106].

### 4.3. Anti-Fatigue

Fatigue is often harmful to biological tissues, which are susceptible to fatigue damage after being subjected to prolonged cyclic loading [107,108]. Fatigue tends to cause degradation of the stiffness of biological tissues by destroying their structure, which in turn causes degradation of biomechanical function [108,109]. It has been shown that excessive mechanical loading can lead to plantar skin lesions, which in turn adversely affect human health [21].

In order to resist the damage caused by fatigue, biological tissues usually have anti-fatigue properties. For example, muscle tissue produces strength and movement through repeated contraction and relaxation, and after prolonged or intense use, it is still able to maintain a certain level of function and performance, delaying the onset of fatigue [110,111]. Ligaments [112,113], tendons [114], and other connective tissues have the mechanical characteristics of high strength and high elasticity, and they are able to withstand large stresses and strains; this mechanical characteristic allows connective tissues to maintain their function under prolonged or high-intensity loads and reduces the risk of fatigue [115].

The heel pad is subjected to severe mechanical conditions, as healthy adults may walk from less than a thousand steps to more than ten thousand steps a day [9]. Under such harsh conditions, the heel pad still maintain structural integrity, still maintaining good cushioning performance after tens of thousands of cyclic loads, so the heel pad has good fatigue characteristics [12].

**Figure 9:**
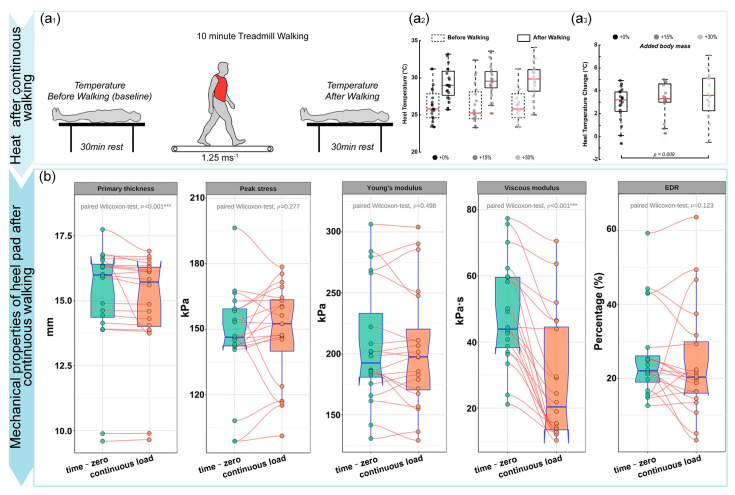
Effect of continuous walking on the mechanical properties of heel pad. (**a1**) Test method. (**a2**) Heat of heel pad after continuous walking. Reproduced with permission from Ref. [9], Copyright 2022, *Frontiers in Bioengineering and Biotechnology*. (**a3**) Heat of heel pad after increased weight bearing, (**b**) Mechanical properties of heel pad after continuous walking. Reproduced with permission from Ref. [31], Copyright 2022, *BMC Musculoskeletal Disorders*, part of Springer Nature. *** *p* < 0.001, two-sided Student’s *t* test.

The mechanical properties of the heel pad after continuous loading were investigated by the human body through continuous walking, and the test process is shown in Figure 9(a1–a3). Under continuous loading, the heel pad tends to increase in energy consumption and temperature, as shown in Figure 9b, and the temperature rise is more obvious with the increase in body weight. The peak strain, peak stress, modulus of elasticity, and EDR of the heel pad did not change significantly before and after cyclic loading, and its thickness and viscous modulus decreased slightly, which also indicated that the heel pad had a strong ability to maintain stable mechanical properties after continuous loading [116,117]. Further, the researchers studied the effect of cyclic loading on the mechanical properties of heel pads in diabetic patients. The results showed that the thickness and viscous modulus of their heel pad also decrease after continuous loading, and the decrease in viscosity means that their cushioning ability decreases, which leads to an increased risk of injury and ulceration of the foot [118]. Fibrous membranes in heel pads are wrapped in almost liquid fat, and the elasticity of heel pads mainly comes from the fibrous membranes, and lesions tend to cause loss of elasticity due to damage to the septum structure, which results in a reduced ability to resist cyclic loading.

### 4.4. Touchdown Stability

Touchdown stability is the ability of the foot to maintain balance and stability during human movement when in touches the ground, and it is critical to a person’s daily activities, movement, and posture control [119,120].

When the heel touches the ground, the hyperelastic heel pad can effectively improve the stability of the touchdown. In the early stage of touching the ground, the soft heel pad can be deformed on the irregular road surface, dispersing the load on the surface of the heel pad and improving the grip of the heel pad [96]; in the late stage of touching the ground, the heel pad is completely compressed, and the stiffness is larger at this time, so it can effectively transfer the load of the foot [94]. At the same time, the heel pad has a certain degree of viscosity. It can avoid the “chatter” and rebound of the heel pad during touchdown, effectively ensuring the touchdown stability [4].

The mechanical anisotropy of the heel pad plays a crucial role in enhancing ground contact stability during human locomotion. During foot–ground interaction, this specialized structure demonstrates dual adaptive mechanisms: vertically, the heel pad absorbs substantial compressive deformation to effectively dissipate impact forces, thereby protecting biological tissues from excessive vertical loading; horizontally, it exhibits controlled tangential motion through constrained displacement within the plantar plane. This dual mechanism simultaneously achieves impact attenuation (via vertical compliance) and slip prevention (through limited tangential displacement [94]), ensuring optimal dynamic stability throughout the stance phase.

## 5. Relationship Between Heel Pad Structure and Materials, Biomechanical Properties, and Their Biomechanical Functions

**Figure 10:**
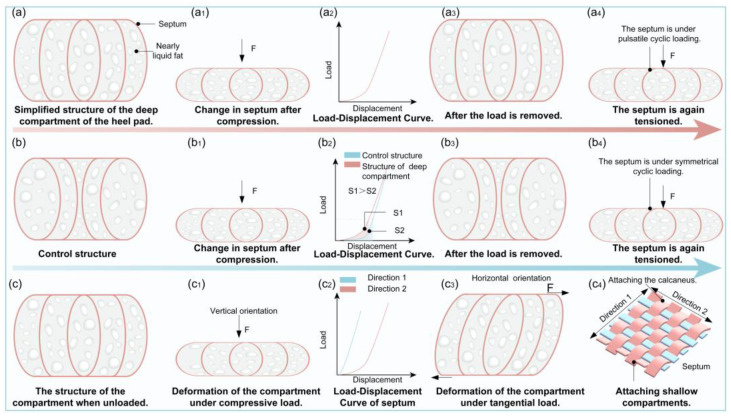
Analysis of the mechanism of compartment structure and fatty material on its multifunctionality. (**a**) The control structure absorbs less impact energy and shows higher stress concentration. (**b**) The deep compartment structure absorbs more energy and offers better cushioning due to septum-fat coupling. (**c**) The septal structure shows anisotropic stiffness, enabling vertical flexibility and horizontal stability for slip resistance.

Harsh mechanical conditions have resulted in a highly specialized structure of the deep compartment of the heel pad. Harsh mechanical conditions mean that the heel pad is subjected to an impact force of 2–3 times the body’s weight when the human body is engaged in strenuous exercise such as running, and the heel pad is subjected to a cyclical load when people walk. In this study, a simplified model of the heel pad compartment structure, as shown in Figure 10a, and a control model of the force deformation are analyzed, in order to analyze the mechanism of the influence of the compartment structure on its multifunctionality. The simplified model of the compartment structure shows that the fat, which is in a liquid state, expands around the compression load, which leads to the septum being stretched and expanding to both sides, as shown in Figure 10(a1). Since the septum consists of collagen and elastic fibers, the stretching of the septum means that the collagen and elastic fibers are stretched, which leads to a nonlinear load–displacement curve of the heel pad under compressive loading due to their nonlinear mechanical properties, as shown in Figure 10(a2).

As for the control structure, as shown in Figure 10b, its internal septum is a concave structure, and the fat of the presented liquid expands to the surrounding area after a compression load, which also leads to the expansion of the septum to the two sides, as shown in Figure 10(b1), and its load–displacement curve is shown in Figure 10(b2). In the early stage of compression, the compression load causes the incompressible liquid to expand outward rapidly, and the fiber membrane on the outermost two sides is then gradually stretched with very little stiffness; in the later stage, the septum is tensioned and entered into the rigid phase, and the control structural stiffness abruptly changes and hardens. Table 4 presents a summary of key research on the structural, biomechanical, and functional characteristics of heel pads.

Therefore, compared with the deep compartment structure, the control structure absorbs less impact energy during the process of deformation. As shown in Figure 10(b2), the deep compartment structure absorbs more energy than the control structure in the large deformation stage, and this kind of septum convex structure and the coupling effect of the fat may be the reason why heel pads have excellent cushioning and vibration damping performance. Compared with the control structure, the convex diaphragm structure is mainly subjected to pulsating cyclic loading, which reduces stress concentration at the junctions and is thus less prone to damage and has a longer service life (see Figure 10(a3,a4,b3,b4). The mechanical anisotropy observed in the heel pad may originate from the inherent anisotropy of its septal architecture. As demonstrated in Figure 10(c2,c4), the septum exhibits superior stiffness along Direction 1 compared to Direction 2. This structural anisotropy facilitates peripheral tissue expansion during vertical compressive loading while maintaining favorable vertical flexibility. Notably, the system demonstrates constrained tangential deformation under horizontal shear stresses, as evidenced in Figure 10(c1,c3). This dual-phase mechanical behavior functionally enhances slip resistance during ground contact by simultaneously permitting vertical energy dissipation and restricting horizontal tissue displacement. Thus, the coupling between the convex structure of the compartment and the viscoelastic fat is an important reason for the mechanical advancement.

**Figure 11:**
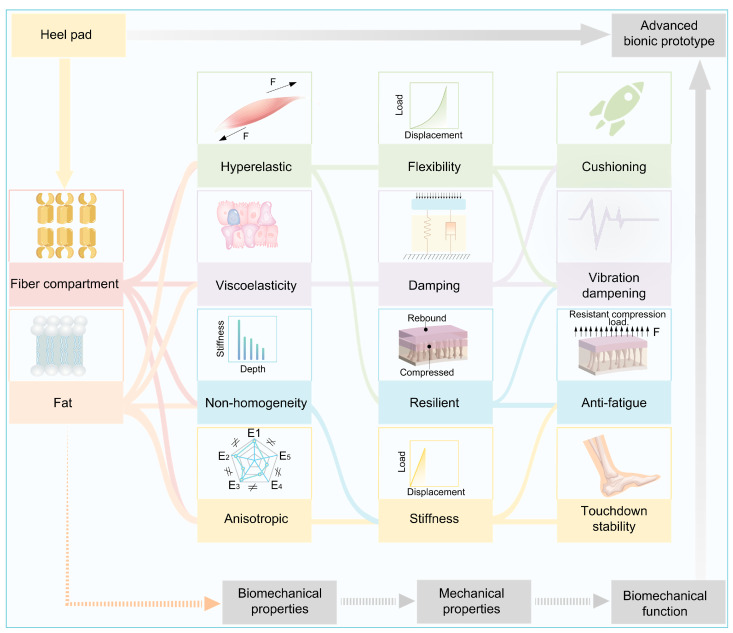
Relationship between heel pad structure and materials, biomechanical properties, and their biomechanical functions.

This study summarizes the relationship between the structure and materials of the heel pad, its biomechanical properties, its mechanical properties, and its biomechanical function, as shown in Figure 11. The heel pad reduces the impact force through sufficient flexibility and viscous damping, reduces the vibration on the human body through damping, and resists the damage caused by fatigue load on the heel pad through sufficient elasticity and stiffness. In addition, it ensures touchdown stability through mechanical anisotropy. Under the coupling of special compartment structure and material, the heel pad is characterized by hyperelasticity, viscoelasticity, non-homogeneity, and anisotropic biomechanical properties, which present mutually exclusive mechanical features, flexibility, damping, resilient, and stiffness, and thus can play the role of cushioning, damping, anti-fatigue, and many other biomechanical functions. Therefore, the advanced mechanical properties of heel pads are attributed to their biomechanical properties, which in turn depend on their compartment structure and material properties, i.e., there is a direct relationship between the structure and materials of heel pad and their advanced mechanical properties. The structural and material composition of heel pads may provide ideal biological prototypes for the preparation of bionic materials with advanced mechanical properties.

This study provides a systematic synthesis of the heel pad’s anatomical architecture, material composition, biomechanical characteristics, and functional dynamics, while concurrently identifying existing research gaps in these domains. The investigation further outlines methodological limitations in current heel pad research and proposes targeted investigative approaches for future studies, with detailed comparative analysis presented in Table 5.

## 6. Perspective of Advanced Materials Based on Heel Pad as Bionic Prototype

**Figure 12:**
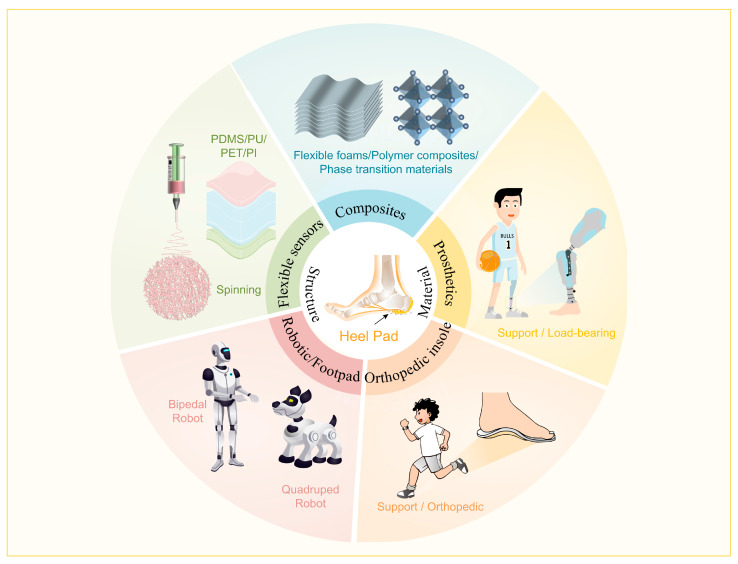
Relevant studies inspired by heel pad and their application areas.

Heel pads with advanced mechanical properties have a wide range of potential applications, and this study outlines the research on advanced materials based on heel pads as bionic prototypes, which include flexible sensors [121,122,123], flexible composites, and some functional materials such as legged robots [124,125,126,127], orthopedic insoles, and prosthetic footplates, as shown in Figure 12.

As an important part of flexible wearable devices, sensors have good flexibility, stretchability, and conductivity, and they can be used as smart wearable devices to monitor and analyze the human body movement and physiological signals in real time, which has received widespread attention [128,129,130,131].

In order to be easy to wear and meet the requirements of flexible electronic devices, flexible sensors must be light, flexible, safe, and with non-toxic properties. Flexible pressure sensors are usually composed of two major parts: the sensing layer and the electrode [132,133,134]. The sensing layer is primarily composed of two essential components, a flexible substrate and an active material, where the careful selection of both substrate and conductive materials plays a critical role in determining the performance of flexible sensors [135,136,137].

These flexible substrates usually do not have multi-functional mechanical properties such as cushioning, anti-fatigue, etc., which can easily be damaged in the process of use due to excessive force or long time of use, especially for pressure sensors applied to the soles of the foot, and multi-functional mechanical properties of the substrate are more required [138,139]. Through the summary of the multifunctionality of the heel pad in this paper, it can be seen that the elastic septum in the compression process of the heel pad is the main part of the impact load, which is interwoven by collagen fibers and elastic fibers, elasticity, and high strength, like a kind of elastic fabric.

Therefore, taking the septum in the heel pad as a bionic prototype, by imitating the arrangement of collagen fibers and elastic fibers within the septum and the material properties of the fibers, a bionic fabric with similar mechanical properties of the septum is prepared, which can be used for the flexible substrate of the sensor to solve the problem of insufficient mechanical properties of the flexible substrate.

Currently, flexible composites are widely used for human body and product protection, particularly for cushioning and vibration damping applications. Common types include flexible foams (e.g., EPS and PU), polymer composites reinforced with nanofillers, phase change materials, and liquid crystal elastomers [140,141,142,143,144]. While flexible foams offer excellent lightweight cushioning, their energy absorption efficiency significantly degrades under repeated impacts [145,146,147]. Additionally, certain rubber and composite materials tend to lose elasticity after prolonged or cyclic loading, which compromises their cushioning and damping capabilities [148,149,150,151,152,153].

In contrast, the human heel pad is capable of withstanding impact forces up to three times its body weight while maintaining superior cushioning, vibration damping, and fatigue resistance. These properties are largely attributed to its unique compartmentalized structure and specialized material composition. Therefore, by adopting the heel pad as a bionic prototype and replicating its structural and material characteristics, it is possible to design advanced flexible composites that overcome the limitations of existing materials.

To enhance the practicality and feasibility of this research, it is recommended to explore potential synthetic materials that exhibit mechanical properties similar to those of the heel pad. Examples include thermoplastic polyurethane (TPU) elastomers, silicone rubber–graphene composites, 3D-printed porous polymer structures, and non-Newtonian shear-thickening materials. These candidates offer promising characteristics in terms of compressive resilience, energy dissipation, and multidirectional mechanical response, making them suitable for the development of multifunctional, fatigue-resistant bionic cushioning systems.

In addition, using the heel pad as a bionic prototype, some functional materials similar to its multifunctionality can be prepared, such as foot pads for improving the stability of robotic touchdowns, orthopedic insoles for the health protection of the human foot, etc. A finite element model (FEM) incorporating a visco-hyperelastic anisotropic material model can be established. The model should be calibrated using experimental data and then used to simulate complex loading scenarios, such as heel strikes during walking or jumping, to analyze the interplay among the three mechanical properties. This integrated approach can help reveal how these properties influence load distribution, energy dissipation, and structural adaptation under real-world biomechanical conditions.

As a professional rehabilitation aid, orthotic insoles play an irreplaceable role in foot health and treatment [154]. As a natural protective and orthopedic pad, the heel pad, with its compartmentalized structure and wrapped viscoelastic fat, effectively ensures the comfort of the human body when walking. Therefore, in the future, the design of orthopedic insoles can consider introducing the structure and material system of heel pad, which may be able to effectively reduce heel pain and improve gait problems by imitating the natural structure and mechanics of the human body.

In the field of prosthetics, the foot plate of prosthetics is mostly made of high-performance carbon fiber material to support, absorb impact, and provide stability [155,156,157]. This material does not have the flexibility and mechanical anisotropy of the human heel pad, resulting in insufficient cushioning performance, difficulty in adapting to complex terrain, and insufficient grip, which leads to poor stability of the ground when walking. In future research on prosthetic limbs, it may be possible to use the heel pad as a bionic prototype to design corresponding flexible bionic material for prosthetic foot plates to improve the stability of movement, achieving self-powered end-of-foot information sensing through triboelectricity [158,159,160] and other mechanisms [161,162]. Furthermore, flexible functional materials are used in many fields such as aerospace and architectural design, and heel pads with advanced mechanical properties can provide design inspiration and an important engine for the development of flexible materials.

## 7. Conclusions and Prospects for Future Research

This review systematically elaborates on the mechanisms by which the structure and material composition of heel pads influence their advanced mechanical properties and functions. It also explores the potential of the heel pad as a bionic prototype and its role in inspiring the study of bionic materials. However, significant challenges remain in thoroughly investigating how the compartmental structure and material composition govern the advanced mechanical functions of the heel pad.

**Figure 13:**
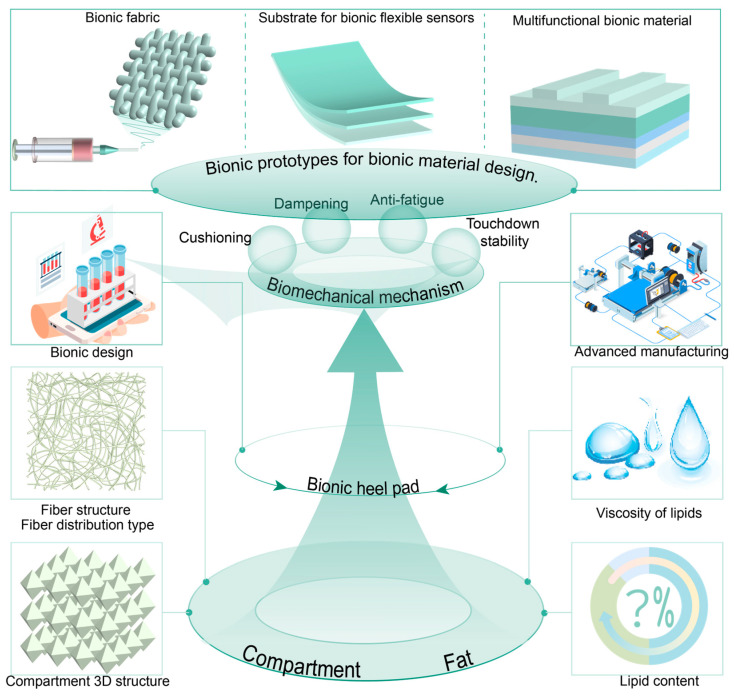
Future research perspectives of heel pad, revealing the biomechanical mechanism of the advanced mechanical properties of heel pad through the integration of cutting-edge technologies such as bionic technology and advanced manufacturing technology.

The microstructure of biological tissues and materials, through coupling, often directly determines their mechanical properties [163]. Based on this principle, future advancements in 3D printing, additive manufacturing, and other advanced manufacturing technologies could enable the development of bionic heel pads with compartment structures and mechanical properties similar to those of natural heel pads. By regulating the compartment structure (e.g., compartment density, concave–convex shapes) and material parameters (e.g., liquid content of fat, fiber content, fat viscosity) of the bionic heel pad, researchers can investigate how these factors influence mechanical properties such as cushioning, vibration damping, anti-fatigue performance, and ground stability. This approach aims to reveal the mechanisms underlying the advanced mechanical properties of the heel pad. The overall technological concept is illustrated in Figure 13.

Since the structure of the heel pad compartment is currently limited to a two-dimensional plane, it is essential to utilize advanced technologies such as Micro-CT and Micro-MR to comprehensively characterize its structure in the future. This includes analyzing the three-dimensional structure of the compartment, the arrangement and properties of collagen and elastic fibers within the septum, the distribution of these fibers, and their mechanical properties, as well as the mechanical properties of the septum itself. These data will provide critical support for the structural design of a bionic heel pad prototype. Additionally, in-depth research on the mechanical properties of fat materials is necessary, including their rheological properties, dynamic viscoelasticity, viscosity, and lipid content. These investigations will provide reliable material parameters to guide the development and preparation of bionic heel pads.

Research on the mechanisms underlying the multi-functionality of the heel pad can provide a solid theoretical foundation for the development of high-performance composite materials and the design of multi-functional flexible materials. This work holds significant scientific importance and potential application value, particularly in advancing the protection of human foot health and improving the diagnosis and treatment of heel-related diseases. 

## Figures and Tables

**Table 2:** Viscoelastic model and parameters of heel pad.

Mathematical Model of Viscoelasticity	Parameters
QLV [66]	$C_{10}\left(MPa\right)=0.1$ $C_{30}\left(MPa\right)=7\left(KPa\right)=7$ $A_{1}\left(1\;ms\right)=0.06$ $A_{2}\left(10\;ms\right)=0.77\;A_{3}\left(0\;ms\right)=0\;A_{4}\left(1\;s\right)=0\;A_{5}\left(10\;s\right)=0.02$
Helmholtz free energy function [67]	$\gamma_{1}(a\;\mathrm{constant})=7.17\times 10^{-1}\;\tau_{1}(s)=6.23\times 10^{-4}$ $\gamma_{2}(a\;\mathrm{constant})=1.55\times 10^{-1}\;\tau_{2}(s)=1.55\times 10^{-2}$ $\gamma_{3}($ $a\;\mathrm{constant})=6.52\times 10^{-2}\;\tau_{3}(s)=9.88\times 10^{4}$ $\gamma_{4}($ $a\;\mathrm{constant})=6.26\times 10^{-2}\;\tau_{4}(s)=9.82\times 10^{5}$
Mooney-Rivlin [31]	$C_{100}\left(Pa\right)=C_{010}\left(Pa\right)=0.01\;C_{110}\left(Pa\right)=0\;C_{200}\left(Pa\right)=C_{020}\left(Pa\right)=0.1$
Maxwell’s model of fifth order [64]	compression rate: 15 mm/s $\;g_{1}\left(MPa\right)=0.19\;\tau_{1}(s)=0.20\;g_{2}\left(MPa\right)=0.11\;\tau_{2}(s)=2.65$ compression rate: 25 mm/s $\;g_{1}\left(MPa\right)=0.18\;\tau_{1}(s)=0.20\;g_{2}\left(MPa\right)=0.11\;\tau_{2}(s)=3.17$ compression rate: 50 mm/s $\;g_{1}\left(MPa\right)=0.17\;\tau_{1}(s)=0.20\;g_{2}\left(MPa\right)=0.11\;\tau_{2}(s)=3.06$ compression rate: 75 mm/s $\;g_{1}=0.16\;\tau_{1}=0.21\;g_{2}=0.12\;\tau_{2}=3.40$(s)
Generalized Rivlin [62]	$C_{10}\left(MPa\right)=4.39\times 10^{-3}$ $C_{20}\left(MPa\right)=6.55\times 10^{-4}\;C_{30}\left(MPa\right)=1.60\times 10^{-3}$ $g_{0}\left(MPa\right)=0.71$ $g_{1}\left(MPa\right)=0.17$ $g_{2}\left(MPa\right)=0.12\;\tau_{1}(s)=0.61(s)$ $\tau_{2}(s)=5.99$
Ogden [63]	c($a\;\mathrm{constant})=9.87\times 10^{-3}$ $C_{30}\left(MPa\right)=1.60\times 10^{-3}\;g_{0}\left(MPa\right)=0.70$ $g_{1}\left(MPa\right)=0.17$ $g_{1}\left(MPa\right)=0.18$ $g_{2}\left(MPa\right)=0.12\;{\;\tau }_{1}(s)=0.57$ $\tau_{2}(s)=6.03$

**Table 3:** Energy dissipation characteristics of heel pad under different test conditions.

In vivo impact test [36]	**Instrument**	**Speed** **/Frequency**	**Energy loss EDR/%**	
	Impact tester	0.72 m/s	77.4	
		0.93 m/s	78.8	
		0.57 m/s	73.8	
		0.94 m/s	73.6	
In vitro test [4]	Instron	2.2 Hz	28.6 ± 6.9 (with calcaneus)	32.3 ± 5.4 (without calcaneus)
Comparison test [105]	Instron	50.4		
	Pendulum	65.5		

**Table 4:** Summary of key research on structural, biomechanical, and functional characteristics of heel pads.

Category	Subcategory/ Research Focus	Author/ Citation	Research Objective	Key Conclusions/Findings
Structure & Material Properties	Anatomical Structure and Composition	Ker [29]	Effects of cyclic loading on compartments	Shorter intervals and higher frequencies increase stiffness
	Age and Pathological Effects	Hsu, T. [35,36]	Impact of aging on collagen/elastic fibers	Elderly heel pads show higher stiffness due to collagen loss and fibrosis
		Hsu, C.C [37,38]	Pathological changes in compartment structure	Thickened/hardened septa reduce cushioning performance
	Thermodynamic Response	Tudor-Locke [9]	Temperature changes during continuous walking	Increased body weight elevates temperature, indicating energy dissipation
Biomechanical Properties	2.1 Nonlinear Stiffness	Miller-Young [31]	Elastic modulus under varying compression speeds	Cutting samples disrupts compartments, lowering modulus (18.4 kPa vs. 2 MPa)
		Ker [29,30]	Differences in in vivo vs. in vitro stiffness	Higher in vivo stiffness due to energy absorption by adjacent tissues
	2.2 Viscoelasticity	Takuo Negishi [64]	Stress relaxation under indentation	Fifth-order Maxwell model fits relaxation curves
		A. N. Natali [52]	Development of visco-hyperelastic constitutive model	Model integrates nonlinearity and time dependence, validated for stress relaxation
		C.G. Fontanella [71]	Quantification of hysteresis	Nonlinear viscoelastic model quantifies energy loss rates
		Robert. Ker [30]	Effect of time intervals on hysteresis	3.7% increase in energy loss rate per 10× interval time increase
Biomechanical Functions	3.1 Cushioning	Kinoshita, H [97]	Age-related cushioning performance	Reduced deformation capacity in elderly leads to higher peak accelerations
	3.2 Vibration Damping	Bennett A N [4]	Energy dissipation in vitro	28.6–32.3% energy loss linked to compartment structure and fat fluidity
		Aerts, P. [105]	Vibration attenuation at resonance	Heel pad absorbs 75–80% energy in 20–35 Hz range, outperforming ankle joints
	3.3 Anti-Fatigue	Qian, Z [12]	Fatigue resistance	Reduced viscous modulus in diabetics increases ulceration risk
	3.4 Touchdown Stability	Chi, K.-J. [94]	Role of mechanical anisotropy	Vertical cushioning + horizontal slip resistance enhance stability

**Table 5:** Inadequacies in research of heel pad and future research methods.

		Inadequacies of Research	Research Methods in the Future
Structure		Lack of 3D modeling of deep large compartments. The arrangement of collagen and elastic fibers in the septum remains to be revealed.	The in vitro heel pad is scanned by micro-CT or micro-MR and modeled by software such as Mimics. The septum obtained by dissecting the heel pad is scanned using electron microscopy to observe the structure and distribution pattern of collagen and elastic fibers.
Materials		Lack of quantification of fat content and septum content. Mechanical properties of fiber septum need to be characterized.	The volume calculation of the modeled deep compartment is done by SolidWorks software 2023. Mechanical properties of the fiber septum obtained by dissection are tested using a universal testing machine.
Biomechanical properties	Hyperelasticity	Since the hyperelasticity of heel pad depends on the deep compartment, hyperelasticity studies of the deep compartment are lacking.	Compression tests are performed on the deep compartment to study its hyperelasticity.
	Viscoelasticity	Lack of research on creep properties of heel pad.	In vitro heel pad tested in combination with testing machine to characterize its creep properties.
	Non-homogeneity	Lack of research on the mechanical properties of heel pad in different positions.	Tests are performed on horizontal section of the heel pad using an ultrasound equipment.
	Anisotropy	Lack of testing of heel pad load–displacement in different directions during human walking.	Combining biplane X-ray transmission system and force plate equipment to test the heel pad in human walking.
Biomechanical function	Cushioning	Lack of cushioning performance testing of in vitro heel pad.	Cushioning performance of in vitro heel pads is tested by an impact tester.
	Vibration damping	Lack of research on the vibration damping properties of in vitro heel pad.	A vibration damping tester need to be constructed using shakers, signal generators, and power amplifiers to test the vibration transfer characteristics of in vitro heel pad.
	Anti-fatigue	The anti-fatigue properties of in vitro heel pad need to be characterized in depth.	The S-N fatigue curves are obtained to characterize the fatigue properties of the in vitro heel pad by applying cyclic loads to it with fatigue testing machine.
	Touchdown stability	Quantification of the relationship between mechanical anisotropy and touchdown stability of heel pad is lacking.	By preparing bionic materials with mechanical properties comparable to heel pad, the experimental study of mechanical anisotropy on touchdown stability is carried out by adjusting the mechanical anisotropy of the bionic materials.

## Data Availability

No new data were created or analyzed in this study. Data sharing is not applicable to this article.
